# Assessing the acceptability and feasibility of encounter decision aids for early stage breast cancer targeted at underserved patients

**DOI:** 10.1186/s12911-016-0384-2

**Published:** 2016-11-21

**Authors:** Shama Alam, Glyn Elwyn, Sanja Percac-Lima, Stuart Grande, Marie-Anne Durand

**Affiliations:** 1 The Dartmouth Institute for Health Policy and Clinical Practice, Level 5, Williamson Translational Research Building, One Medical Center Drive, Lebanon, NH 03756 USA; 2 Massachusetts General Hospital, Boston, MA USA; 3 Harvard Medical School, Boston, MA USA

**Keywords:** Decision aids, Encounter decision aids, Health disparities, Breast cancer, Low socioeconomic status, Acceptability, Feasibility

## Abstract

**Background:**

Women of low socioeconomic status (SES) diagnosed with early stage breast cancer are less likely to be involved in treatment decisions. They tend to report higher decisional regret and poorer communication. Evidence suggests that well-designed encounter decision aids (DAs) could improve outcomes and potentially reduce healthcare disparities. Our goal was to evaluate the acceptability and feasibility of encounter decision aids (Option Grid, Comic Option Grid, and Picture Option Grid) adapted for a low-SES and low-literacy population.

**Methods:**

We used a multi-phase, mixed-methods approach. In phase 1, we conducted a focus group with rural community stakeholders. In phase 2, we developed and administered a web-based questionnaire with patients of low and high SES. In phase 3, we interviewed patients of low SES and relevant healthcare professionals.

**Results:**

Data from phase 1 (*n* = 5) highlighted the importance of addressing treatment costs for patients. Data from phase 2 (*n* = 268) and phase 3 (*n* = 15) indicated that using both visual displays and numbers are helpful for understanding statistical information. Data from all three phases suggested that using plain language and simple images (Picture Option Grid) was most acceptable and feasible. The Comic Option Grid was deemed least acceptable.

**Conclusion:**

Option Grid and Picture Option Grid appeared acceptable and feasible in facilitating patient involvement and improving perceived understanding among patients of high and low SES. Picture Option Grid was considered most acceptable, accessible and feasible in the clinic visit. However, given the small sample sizes used, those findings need to be interpreted with caution. Further research is needed to determine the impact of pictorial and text-based encounter decision aids in underserved patients and across socioeconomic strata.

## Background

Despite advances in early stage breast cancer treatment, variation in outcomes persists with underserved patients experiencing poorer communication, poorer engagement in decision making, poorer outcomes, and higher mortality rates [1, 2]. Low socioeconomic status (SES), as measured by Medicaid status and census data is a risk factor for unfavorable breast cancer outcomes, irrespective of race or ethnicity [3]. Women of lower SES face a disproportionate quality of life burden from an early stage breast cancer diagnosis compared with women from other socioeconomic groups [4]. Research reveals that women of low SES with early stage breast cancer are less likely to be involved in decision making and to experience supportive doctor-patient communication [5, 6]. They are thus more likely to make uninformed decisions, to be unaware of the harms and benefits of available options, and to fail to exert their personal preferences.

It remains unclear, however, how best to promote participation in decision making, improve knowledge, and reduce disparities in patients of low SES diagnosed with early stage breast cancer [7–9]. A systematic review of decision aids for early stage breast cancer showed that decision aids increased patients’ knowledge, decreased patients’ decisional conflict, and increased patients’ satisfaction with the decision making process [10]. Decision aids are interventions designed to facilitate shared decision making and patient participation in preference-sensitive health care decisions. Decision aids are delivered using diverse formats (e.g., web-based, video, audio, print), and have been shown to be effective in controlled contexts. However, their implementation in routine clinical settings remains difficult, partly due to a lack of attention to end-user acceptability [11]. Is it possible to design patient decision aids that are acceptable, feasible, and beneficial to underserved patients? Houts et al. [12] examined the role of simple language and images on health communication, suggesting that combining images and plain language increases patient retention, comprehension, recall, and adherence, and can be especially helpful to patients with lower textual literacy. Durand’s systematic review and meta-analysis also indicate that decision aids adapted for underserved patients significantly improved outcomes in patients with lower SES and lower literacy [9].

To date, most decision aids have been introduced to patients *ahead* of clinical encounters (i.e., *pre-encounter* decision aids). They typically provide extensive information, often on the Internet, with poor accessibility and readability. A systematic review of the readability and cultural sensitivity of web-based decision aids for cancer screening and treatment indicated that the vast majority of decision aids had low readability, complicated text, and a lack of cultural sensitivity [13]. Further, research has shown that although decision aids improve outcomes in controlled settings (with literate audiences), their use in routine care remains rare because of resistance to implementation [14].

Shorter, simpler decision aids designed for use in clinical encounters (encounter decision aids) have received less attention than complex pre-encounter interventions [15]. An encounter decision aid provides evidence-based information about significant harms and benefits of available options, is used in the clinic visit to facilitate the elicitation of patient values and preferences, and enables clinicians to tailor information to patients’ needs and characteristics. They have been shown to increase patients’ knowledge and patient participation in decision making, to improve risk perception, and in some instances, to influence choice and improve adherence to treatments [16–21].

The aim of this study was to assess the acceptability and feasibility of three encounter decision aids (one text-based encounter decision aid and two pictorial encounter decisions aids) targeted at a low SES and low literacy population using plain language and images.

## Methods

The study was divided into three phases: (i) focus group with rural community stakeholders; (ii) completion of a web-based questionnaire with women of low and high SES diagnosed with early-stage breast cancer between 2009 and 2015, and (iii) semi-structured interviews with women of low SES who have had early stage breast cancer and with healthcare professionals.

### Encounter decision aid development

**Fig. 1:**
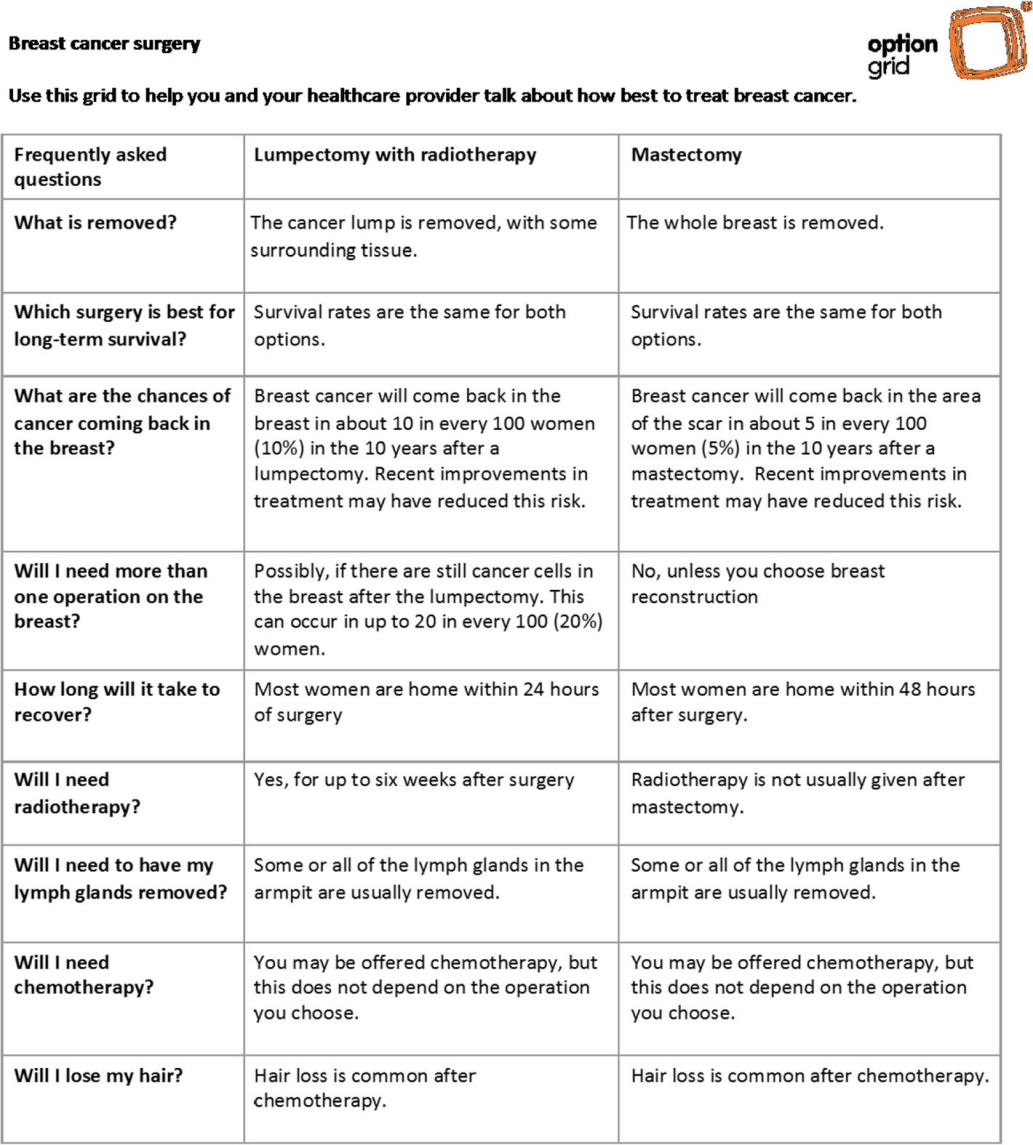
Option Grid decision aid: Breast cancer surgery

**Fig. 2:**
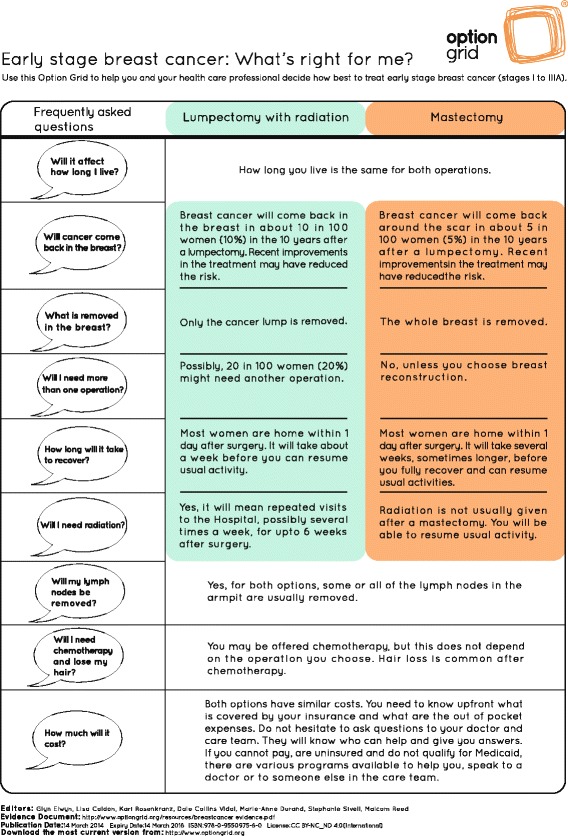
Option Grid decision aid: Early stage breast cancer: What's right for me?

Option Grid™ decision aids for clinical encounters were first developed in 2010. The Option Grid decision aid (Fig. 1) for early-stage breast cancer was derived from a long-form, web-based decision aid, shown to facilitate readiness to decide and strengthen surgery intentions [22, 23]. An Option Grid decision aid is a one-page, evidence-based summary of available options presented in a tabular format, listing the frequently asked questions (FAQs) that patients normally consider when making treatment decisions for early stage breast cancer (www.optiongrid.org). The Option Grid decision aid used in this study was adapted for women of low SES, to include colors, improve the layout, and simplify the language (Fig. 2).

**Fig. 3:**
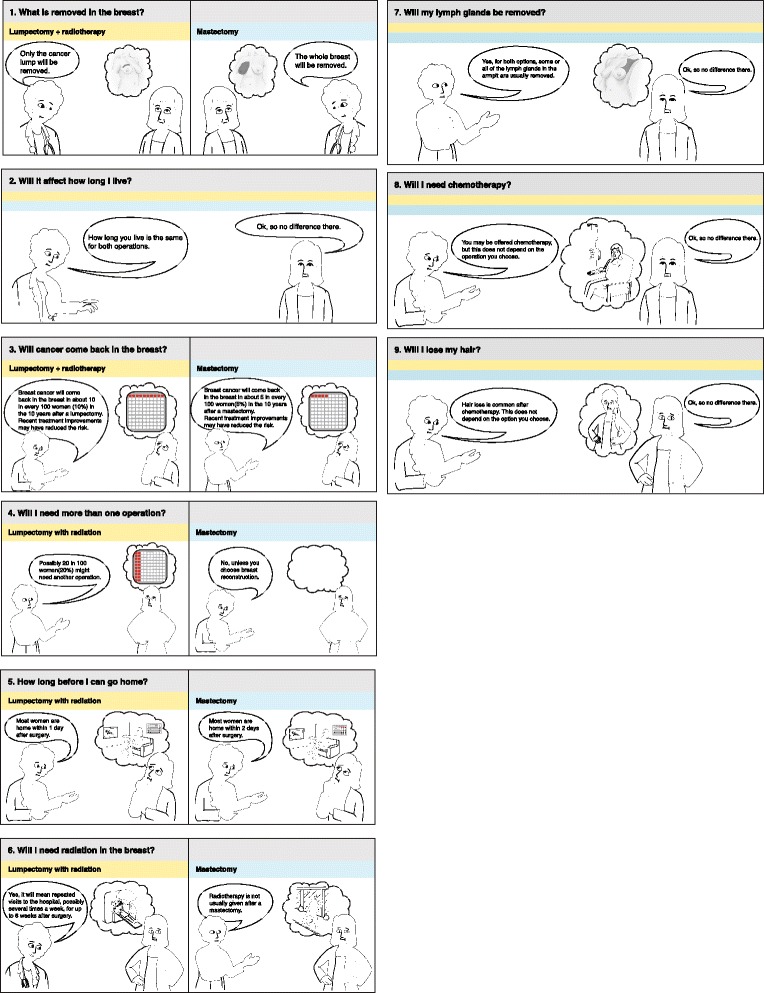
Comic Option Grid decision aid: Early stage breast cancer: What's right for me?

**Fig. 4:**
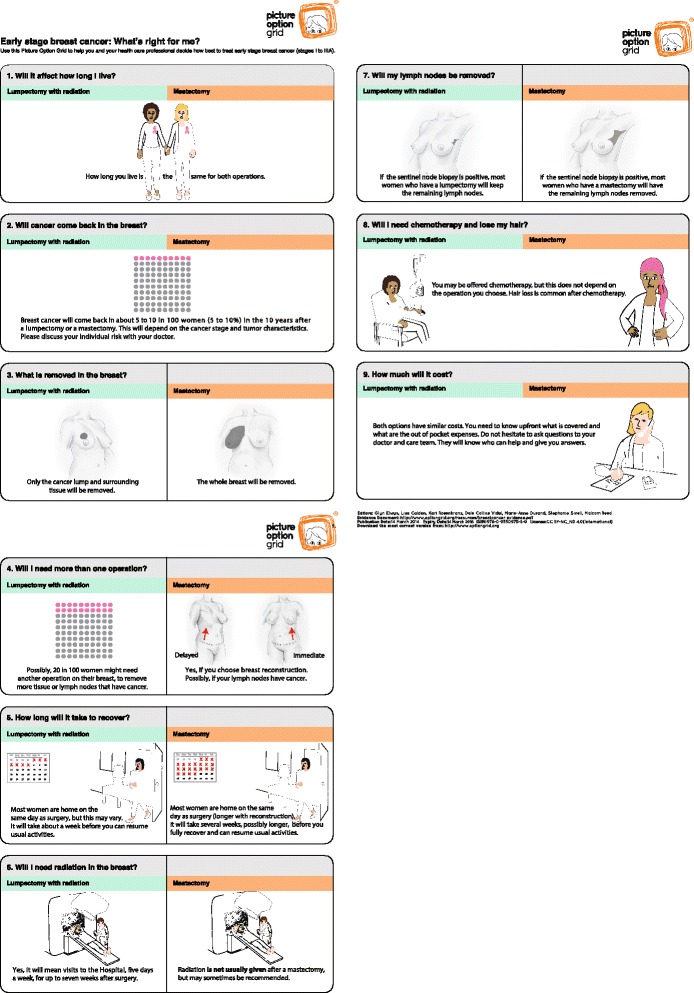
Picture Option Grid decision aid: Early stage breast cancer: What's right for me?

Comic Option Grid (Fig. 3) and Picture Option Grid (Fig. 4) are derived from the Option Grid decision aid. They use the same evidence and include images that illustrate the answers to the FAQs. Prototypes of the pictorial encounter decision aids were initially developed and tested using community-based participatory research (CBPR) [24]. All three encounter decision aids are paper-based, ranging from one to three pages in length, and can be read in less than five minutes with a Flesh-Kincaid grade level of 6.7.

### Setting and participants

In phase 1, a convenience sample of rural community stakeholders was identified by breast surgeons at the Norris Cotton Cancer Center (NCCC) (Lebanon, New Hampshire). Community stakeholders were defined as individuals that can affect a decision with a vested interest in reducing healthcare disparities for low SES women. Potential community stakeholders may range from academic institutions and hospitals to elected officials. The rationale for including rural community stakeholders in phase 1 was to elicit feedback from stakeholders within the community who interact and work with low SES groups in the Upper Valley in New Hampshire and Vermont. In phase 2, we recruited online participants through Facebook advertising and Qualtrics Panels. Qualtrics is an online service that worked with our research group to build a panel with specific requirements regarding sample size, target demographics, survey complexity, and survey length. We included women aged 18 years or older who had been diagnosed with early stage breast cancer (I to IIIA) between 2009 and 2015. We included women of high SES and women who were on Medicaid or Medicare without supplemental insurance (a proxy for low SES) and women who were uninsured. We excluded women who had not yet started breast cancer treatments. In phase 3, a convenience sample of women of low SES who were on Medicaid or Medicare without supplemental insurance or without health insurance recently diagnosed with early stage breast cancer, was identified by clinicians at the Dartmouth-Hitchcock Medical Center (Lebanon, NH) and Chelsea HealthCare Center (Chelsea, MA). We also encouraged participation from health professionals with expertise in breast cancer and shared decision making and recruited a convenience sample of these health professionals from Dartmouth-Hitchcock Medical Center and Chelsea HealthCare Center. Participants were recruited between January and August 2015.

### Data collection

In phase 1, we conducted a focus group and audio-recorded the session. In phase 2, a web-based questionnaire, hosted on Qualtrics, was developed and piloted with academics and members of the public to refine wording. Participants provided demographic characteristics and were randomly allocated to view one of four questionnaires and interventions: Option Grid decision aid (19-items), Comic Option Grid (21-items), Picture Option Grid (21-items), or a comparison of Comic Option Grid and Picture Option Grid (29-items). In phase 3, semi-structured interviews of patients and healthcare professionals were conducted and audio-recorded. In phases 1-3, the focus group discussion, the web-based questionnaire, and semi-structured interviews explored comprehension of health information, visual acuity, layout, usability, acceptability and feasibility of all three encounter decision aids, and aimed to determine the participant’s preference for one of the encounter decision aids.

### Analysis

For the web-based questionnaire, we used descriptive statistics, the chi-square measure of association, and correlation analyses using Kendall Tau B. Analyses were conducted using STATA. Semi-structured interview and focus group transcripts were analyzed using thematic analysis in ATLAS.ti. Inductive coding techniques and the constant comparison method were used. Two investigators (M-AD, SA) reached consensus on codes and definitions, setting priori rules for coding salient themes. Discrepancies were resolved through discussions of coding decisions among study team members.

## Results

### Phase 1

We reached out to seven rural community stakeholders and recruited five stakeholders for the focus group: one parish nurse (registered nurse specialist within a faith community), one pastoral care coordinator, one advanced practice clinician (nurse-practitioner working with cancer survivors in the Upper Valley, NH, VT), a continuing care manager in cancer care, and the executive director of a local non-profit organization supporting homeless families. The stakeholders felt that the layout of Comic Option Grid was crowded and confusing, making it more difficult to read. Four participants felt that Comic Option Grid was patronizing and overly simplified. These comments reflect similar findings from a previous study using CBPR [24]. All participants agreed that the topics of treatment costs and recovery time were paramount and should be included, especially when targeting patients of low SES. Overall, the community stakeholders considered that an encounter decision aid would facilitate a dialogue between patients and clinicians, and promote informed choice. Four out of five participants preferred Picture Option Grid.

### Phase 2

There were 780,000 US resident women ages 18–65+ on Facebook that met the eligibility criteria. Five advertisements resulted in 32,572 impressions with a reach of 20,517 women. Of the women reached, 176 women (0.86%) clicked the ad. Of the women who clicked the ad, 49 women (28%) proceeded past the study information page. In using Qualtrics Panels, there were approximately 2400 US resident women ages 18–65+ who also met the eligibility criteria and were invited to complete the web-based questionnaire through a Qualtrics panel. 219 women (9%) proceeded past the study information page.

A total of 268 individuals responded to the survey. Of the respondents, 211 (79%) respondents were between the ages of 45—74 years. A description of respondent characteristics can be found in Table 1. In total, 249 out of 268 respondents (96%) answered all questions (Table 2). A Kendall's tau-b correlation was conducted to determine the relationship between encounter decision aid preference and 1) health insurance and 2) education level. There appears to be a positive correlation between health insurance status and encounter decision aid preference, however this is not statistically significant (*τ*_b_ = 0.527, *p* = 0.344). There also appears to be a positive correlation between education level and encounter decision aid preference, however this is not statistically significant (*τ*_b_ = 0.755, *p* = 0.691).

**Table 1:** Patient characteristics (Phases 2 and 3)

Characteristics	Categories	Interviews (*n* = 10)		Web-based Questionnaire (*n* = 268)	
		n	%	n	%
Age	18-24	0	0.0	4	1.5
	25-34	1	10.0	17	6.3
	35-44	1	10.0	22	8.2
	45-54	1	10.0	65	24.3
	55-64	4	40.0	68	25.4
	65-74	2	20.0	78	29.1
	75 years or older	1	10.0	14	5.2
Breast Cancer Stage	Stage 1	4	40.0	146	54.5
	Stage 2	3	30.0	54	20.1
	Stage 3A	1	10.0	11	4.1
	Stage 3B	1	10.0	10	3.7
	Stage 3C	1	10.0	5	1.9
	I don’t know	0	0.0	21	7.8
	Missing	0	0.0	21	7.8
Ethnicity	Hispanic or Latino	1	10.0	18	6.7
	Not Hispanic or Latino	9	90.0	232	86.6
	Missing	0	0.0	18	6.7
Race	American Indian or Alaska Native	0	0.0	0	0.0
	Asian	0	0.0	10	3.7
	Black or African American	0	0.0	16	6.0
	White or Caucasian	10	100.0	223	83.2
	Missing	0	0.0	19	7.1
Education completed	Elementary	0	0.0	1	0.4
	Some high school	5	50.0	3	1.1
	High school graduate	2	20.0	61	22.8
	Some college or technical school	2	20.0	73	27.2
	College graduate	0	0.0	65	24.3
	Graduate school	1	10.0	48	17.9
	Missing	0	0.0	17	6.3
Health Insurance Coverage	Private	0	0.0	131	48.9
	Medicaid or Medicare (w/o supplemental insurance)	10	100.0	110	41.0
	Uninsured	0	0.0	8	3.0
	Missing	0	0.0	19	7.1

**Table 2:** Online respondents’ views on comprehension of health information, layout, acceptability and feasibility of encounter decision aids, stratified by SES

Statement	Option grid			Comic option grid			Picture option grid		
	(*n* = 62)			(*n* = 127)^a^			(*n* = 124)^a^		
	n^b^			n^b^			n^b^		
	(%)			(%)			(%)		
	All SES	Low SES	High SES	All SES	Low SES	High SES	All SES	Low SES	High SES
I like the look and feel of …	45 (72.6)	22 (35.5)	23 (37.1)	67 (52.8)	33 (26.0)	34 (26.8)	89 (71.8)	44 (35.5)	45 (36.3)
The layout is not confusing	43 (69.4)	22 (35.5)	21 (33.9)	74 (58.3)	33 (26.0)	41 (32.3)	93 (75.0)	44 (35.5)	49 (39.5)
I understand all the information included	49 (79.0)	24 (38.7)	25 (40.3)	103 (81.1)	46 (36.2)	57 (44.9)	102 (82.2)	49 (39.5)	53 (42.7)
Using pictures is helpful	NA			73 (57.4)	36 (28.3)	37 (29.1)	94 (75.8)	49 (39.5)	45 (36.3)
The pictures help me understand the textual content	NA			72 (56.7)	32 (25.2)	40 (31.5)	95 (76.6)	50 (40.3)	45 (36.3)
I like the cartoon characters	NA			62 (48.8)	31 (24.4)	31 (24.4)	NA		
The pictograms are not confusing	NA			59 (46.5)	26 (20.5)	33 (26.0)	70 (56.5)	29 (23.4)	41 (33.1)
I like the order of the frequently asked questions	47 (75.8)	25 (40.3)	22 (35.5)	88 (69.3)	43 (33.9)	45 (35.4)	98 (79.0)	48 (38.7)	50 (40.3)
I find this tool very helpful	47 (75.8)	24 (38.7)	23 (37.1)	68 (53.5)	32 (25.2)	36 (28.3)	86 (69.4)	40 (32.3)	46 (37.1)
I would recommend this tool to other women who have been diagnosed with early stage breast cancer	45 (72.6)	23 (37.1)	22 (35.5)	80 (63.0)	41 (32.3)	39 (30.7)	96 (77.4)	47 (37.9)	49 (39.5)

*SES* socio-economic status (using health insurance status as a proxy), *NA* not applicable; ^a^268 respondents were randomly allocated to one of four surveys looking at Option Grid, Comic Option Grid, Picture Option Grid, and Comic Option Grid versus Picture Option Grid. 64 respondents viewed the Comic Option Grid versus Picture Option Grid survey and provided comments on both encounter decision aids; ^b^Values and percentages represent respondents that answered, “agree” and “strongly agree” with statements

### Option Grid

Sixty two participants were randomly allocated to view Option Grid. Most respondents (>69%) liked the layout and design of Option Grid, and understood all information provided (79%). Most respondents found the tool helpful and would recommend Option Grid to other women diagnosed with early stage breast cancer (Table 2). In addition, 29 respondents (47%) provided free text comments and indicated that Option Grid was an appropriate resource for a patient who had been newly diagnosed with early stage breast cancer. Respondents felt that Option Grid was brief but concise enough to provide essential information required to facilitate further discussions and informed treatment decisions. Respondents reacted positively to the simplified Option Grid. They considered that the intervention represented an initial, helpful resource for women diagnosed with early stage breast cancer. Many recalled their own experience of receiving a diagnosis of early stage breast cancer as overwhelming and confusing. In addition to reviewing the Option Grid, respondents were asked to select the tool they preferred by viewing snapshots of Option Grid, Comic Option Grid, and Picture Option Grid. Out of the 62 respondents randomly allocated to review Option Grid, 53% selected Option Grid as the preferred intervention. When respondents were asked to rate Option Grid on a scale of 1-5 (1 being lowest and 5 highest), the respondents’ mean score was 4.1 (SE 0.13).

### Comic option grid

In total, 127 participants were randomly allocated to view Comic Option Grid. Only 52.8% of participants liked the design and layout (58.3%) of Comic Option Grid. Although 81% understood all information provided, 43% found the images unhelpful. Over half of all respondents (51.2%) disliked the use of cartoon characters. Although 103 respondents (81%) understood all the information provided, only 59 respondents (47%) understood the reoccurrence rates depicted in the pictograms (Table 2). In addition, 84 respondents (66%) provided comments. They felt that the use of cartoon characters in Comic Option Grid was insensitive, trivialized the patient experience, and was neither diverse nor realistic enough. One respondent stated that she found ‘*the whole thing a little flippant, as this is really a serious conversation to be having with a surgeon’.* In addition to reviewing the Comic Option Grid, respondents were asked to select the intervention they preferred by viewing snapshots of the Option Grid, Comic Option Grid, and Picture Option Grid. Out of the 127 respondents randomly allocated to review Comic Option Grid, 33% selected Comic Option Grid as the tool they preferred. When respondents were asked to rate Comic Option Grid on a scale of 1-5 (1 being lowest and 5 highest), the respondents’ mean score was 3.7 (SE 0.14).

### Picture option grid

In total, 124 participants were randomly allocated to view the Picture Option Grid. The majority of respondents (>65%) liked the layout of Picture Option Grid, understood the information provided (82.2%), and found the use of pictures helpful (75.8%). Although 77% of respondents found images helpful in understanding textual content, only 55% reported understanding the cancer recurrence rates depicted in the pictograms. Most respondents found this tool helpful and would recommend Picture Option Grid to other women diagnosed with early stage breast cancer (77.4%). In addition, 60 respondents (48%) provided comments. They reported that Picture Option Grid was accessible, helpful and straightforward. Similar to the simplified Option Grid, respondents felt that the use of images and concise information would contribute to facilitating further discussions with their clinicians and inform their treatment decisions. One respondent provided the following statement, ‘*the Picture [Option] Grid gives straight answers when the brain is trying to process - Yes, and I have Cancer.’* Various respondents found the combination of images and text helpful. In addition to reviewing the Picture Option Grid, respondents were asked to select the tool they preferred by viewing snapshots of the Option Grid, Comic Option Grid, and Picture Option Grid. Out of the 124 respondents randomly allocated to review Picture Option Grid, 52% selected Picture Option Grid as the tool they preferred. When asked to rate it on a scale of 1-5 (1 being lowest and 5 highest), the respondents’ mean score was 4.2 (SE 0.13).

When all respondents were asked to select their preferred encounter decision aid, 34% preferred Picture Option Grid, 23% preferred Option Grid, and 21% selected Comic Option Grid. The remaining participants did not answer this question.

### Phase 3

We recruited five healthcare professionals: two breast surgeons, one reconstructive surgeon, one primary care clinician, and a patient support executive. Healthcare professionals interviews averaged 27 min and were conducted face to face. All participants preferred Picture Option Grid.

We reached out to 26 women and recruited 10 women of low SES who had been diagnosed with early stage breast cancer in the past five years. The mean age was 56.8 years (SE 4.40). Patient interviews averaged 31 min. Six interviews were conducted via telephone and four interviews were conducted face-to-face. A description of patients’ characteristics can be found in Table 1. When all participants were asked to select their preferred encounter decision aid, 13 participants out of 15 (86%) selected Picture Option Grid. The following themes emerged, and are summarized in Table 3: Purpose of the encounter decision aids, Benefits of the encounter decision aids, and Feasibility of the encounter decision aids.

**Table 3:** Themes identified in interviews

Themes	Sub-themes
Purpose of the encounter decision aids	Preparing and informing patients
	Complete overview of information
Benefits of the encounter decision aids	Pictorial superiority
	Realistic portrayal of the treatment process
	Language accessibility
	Promotes engagement in decision making
Feasibility of the encounter decision aids	Post-diagnosis
	Before or during the surgical consultation
	Beneficial for all

### Purpose of the encounter decision aids

Eight out of ten patient participants and all healthcare professionals observed that all three encounter decision aids aimed to prepare and inform patients of available treatment options for early-stage breast cancer, specifically with the use of realistic images in the Picture Option Grid.

*“It just kind of gives you an idea, puts you there, you know.”* [Patient]

The majority of patient participants felt that the images would have prepared them for the treatment and recovery period, and would have alleviated some of their fears.

Eight out of ten patient participants considered that all three encounter decision aids provided a complete overview of information, analogous to their own discussion with the surgeon.

*“It's a simple question and answer, and many of the questions that I asked when I went in initially.”* [Patient]

*“I think those* [FAQs] *are great questions […], and this would’ve helped me a lot.*” [Patient]

### Benefits of the encounter decision aids

Nine out of ten patient participants highlighted the benefits of images in facilitating and understanding health information. They felt that images realistically portrayed important and detailed information while simplifying information processing. By stating that realistic images helped them understand the textual content, the patients were thus referring, in their own words, to pictorial superiority. All five healthcare professionals felt that the images used in Comic Option Grid and Picture Option Grid would improve understanding of health information for all patients, irrespective of SES, but particularly for patients with low literacy and limited English proficiency.

*“I sometimes think when people come in and they have someone talking to them, they don't always hear everything they say, and I think seeing a visual sometimes is more helpful.”* [Patient]

*“The pictures really show us what's going on…it's very straightforward.”* [Healthcare professional]

Both patient participants and healthcare professionals reported that the benefits of plain language (Option Grid) and images (Comic Option Grid and Picture Option Grid) are even greater with newly diagnosed patients who are overwhelmed by their cancer diagnosis and may struggle to process and remember information.

*“It's easy to read. Easy to look at […] the way it's set up seems very clear. “*[Patient]

*“I think it will influence the women who are so overwhelmed that either they’re not reading what we send them, or not watching the video…”* [Healthcare professional]

Six patient participants and all healthcare professionals considered that another benefit of the encounter decision aids was to promote patient engagement in decision making. The Option Grid and the Picture Option Grid were perceived to help patients formulate and ask questions, improve confidence, and clarify their treatment preferences.

*“I think if someone has looked at it ahead of time, then they will already have an idea of what questions they want to ask. So that will influence how the conversation goes.”* [Healthcare professional]

### Feasibility of the encounter decision aids

All participants (patients and healthcare professionals) felt that the Option Grid and Picture Option Grid were feasible and highly usable in the clinic visit and would benefit a wide range of women diagnosed with early stage breast cancer, irrespective of literacy and SES. Comic Option Grid was deemed least usable and feasible.

Six patient participants and all healthcare professionals felt that offering the Option Grid and Picture Option Grid routinely was feasible and would be particularly beneficial if provided post-diagnosis (e.g., mailed to patients in advance). They also felt that it would be very helpful to use the Picture Option Grid in the consultation, to guide their questions and discussions with the surgeon.

*“So if it was given to me at the time of diagnosis and then I had the appointment with the surgeon to discuss all of this, then it would give me a better idea of what questions to ask.”* [Patient]

“*If I used option grids in my practice, I would really want the patients to have had a chance to get them in advance.”* [Healthcare professional]

Overall, the majority of participants preferred Picture Option Grid. All patient participants stated that they would recommend the Picture Option Grid to other newly diagnosed breast cancer patients.

## Discussion

The study findings indicate that Option Grid and Picture Option Grid appeared acceptable and feasible in-clinic, for women of low and high SES. They were perceived to facilitate shared decision-making in early-stage breast cancer treatment decisions, especially among patients with limited health literacy and limited English proficiency. The perceived insensitivity of the cartoon characters used in Comic Option Grid affected its acceptability. Picture Option Grid was considered most acceptable, accessible, and feasible in the clinic visit across all participants and phases of the study. Most participants (both patients and healthcare professionals) felt that the conciseness of the information provided in the encounter decision aids and the use of plain language on its own (Option Grid), or in conjunction with simple images (Picture Option Grid) clarified information, and facilitated perceived understanding. Picture Option Grid included information on costs associated with treatment type and recovery time and also helped patients prepare for the upcoming treatments while engaging in an informed discussion about treatment options with their clinician. Some patients reported difficulties understanding the outcome probabilities and icon arrays representing the risk of cancer recurrence.

The strengths of the study included a mixed-methods approach of collecting feedback from a variety of stakeholders in several phases of testing. The mixed-methods approach provides stronger concluding evidence through convergence and corroboration of research findings [25]. A potential limitation of this study includes self-selection bias, which is a recognized limitation of web-based survey research [26]. In any given Internet community, some individuals are more likely than others to complete a web-based survey [27, 28]. An additional limitation of this study is that earlier versions of Option Grid and Comic Option Grid did not provide information specific to low SES women, such as costs associated with treatment type and recovery time. The rural community stakeholders recommended including these points in subsequent versions of the encounter decision aids that were viewed in the phase 2 web-based questionnaire and phase 3 interviews. Potential limitations include the small sample size of the web-based survey and a small number of participants of low SES, lower educational attainment, and diverse race and ethnicity. Although we targeted women of low SES (on Medicaid or Medicare without supplemental insurance or no insurance), a large proportion of the web-based questionnaire respondents were of a higher educational attainment and only 44% of respondents had either public insurance or no insurance. The majority of participants identified as White or Caucasian. The population captured in this study is representative of the population where early stage breast cancer’s incidence is highest: Caucasian women. However, given the widening gap in mortality between African-American women and Caucasian women, further research in developing accessible and culturally appropriate encounter decision aids needs to be undertaken with African-American patients of varying SES.

The majority of participants from the three study phases felt images accurately conveyed significant and detailed information while simplifying information processing for the patient. Participants in all three study phases reported that the benefits of the images were even greater when patients were anxious and emotional, as a result of a recent cancer diagnosis, and unable to process information rationally and efficiently.

Our study suggests that a sizeable proportion of participants struggled to comprehend numerical estimates of risk associated with cancer recurrence, even when presented as icon arrays. Existing literature on numeracy corroborates our findings [29–31]. Although visual displays are helpful in understanding statistical health information for people with low numeracy [29, 30], people who lack graph literacy may find numbers alone easier to process [31]. While gauging patients’ numeracy skills in the clinical setting may be difficult, it is essential to design decision aids that will benefit patients of varying literacy and numeracy skills, and teach medical or other personnel to help patients of lower graphic numeracy process this information adequately [32].

Our findings indicate that encounter decision aids (Option Grid decision aid and Picture Option Grid) seem acceptable and feasible for early stage breast cancer, for women of varying socioeconomic status and health professionals included in our sample. This is consistent with existing literature on the acceptability and benefits of encounter decision aids [16, 17, 19]. However, our study is the first to explore the acceptability and feasibility of encounter decision aids (incorporating plain language and pictures) targeted at women of low SES. The combination of simple language and images was perceived to facilitate information processing and was acceptable to both women of high and low SES [33, 34].

## Conclusion

Feedback from patients of varying SES (including a sizeable proportion of women of low SES), clinicians, and stakeholders demonstrate that encounter decision aids seem accessible, acceptable, and feasible within the clinic encounter. An encounter decision aid that uses plain language and incorporates images to help patients visualize available treatment options and prepare them for upcoming treatments was deemed most acceptable and feasible in the clinic visit. However, given the small sample sizes used in this study, further research is needed to determine the impact of pictorial and text-based encounter decision aids in underserved patients, and across socioeconomic strata.

Participants across all socioeconomic strata considered the combined use of plain language and images in Picture Option Grid acceptable and feasible in the clinic visit. The finding that a large proportion of participants, irrespective of SES, had difficulties grasping numerical estimates of risk has implications for how patients with lower educational attainment, literacy, and numeracy conceptualize risk of cancer reoccurrence. It is important that health care providers acknowledge that a patient’s educational attainment, literacy, and numeracy skills may impact their treatment choice when counseling patients [35]. If patients are confused by numeracy concepts, it may limit their awareness of the options available to them and restrict their ability to express their preferences and concerns. Patients may benefit from receiving a simplified encounter decision aid intervention post-diagnosis. Early decision support using simplified encounter decision aids may increase the opportunity to gain knowledge and clarify personal values in preparation for engagement in shared decision making. More research is needed to determine the effectiveness of Picture Option Grid in women of low SES and across socioeconomic strata. This study and others [8, 10] provide policy implications. It is becoming clear that the development and delivery of patient information needs to pay special attention to user-centered design and adopt methods that reduce numeracy and literacy barriers as much as is feasible. New research in risk communication techniques is reinforcing the need to use visualizations, easy to grasp comparisons and, where possible, interactive methods to engage attention [36, 37].

## Abbreviations

CBPR: Community-based participatory research
DA: Decision aid
FAQ: Frequently asked questions
SES: Socioeconomic status
